# 3D-Printed Conformal Meta-Lens with Multiple Beam-Shaping Functionalities for Mm-Wave Sensing Applications †

**DOI:** 10.3390/s24092826

**Published:** 2024-04-29

**Authors:** Noureddine Melouki, Fahad Ahmed, Peyman PourMohammadi, Hassan Naseri, Mohamed Sedigh Bizan, Amjad Iqbal, Tayeb A. Denidni

**Affiliations:** Centre-Energie Matériaux et Télécommunications, Institut National de la Recherche Scientifique, Montreal, QC H5A 1K6, Canada; ahmed.fahad@inrs.ca (F.A.); peyman.pourmohammadi@inrs.ca (P.P.); hassan.naseri.gheisanab@inrs.ca (H.N.); mohamed.bizan@inrs.ca (M.S.B.); amjad.iqbal@inrs.ca (A.I.); tayeb.denidni@inrs.ca (T.A.D.)

**Keywords:** metamaterial, genetic algorithm (GA), 3D printing, conformal, beam deflection, orbital angular momentum (OAM), transmitarray (TA), millimeter-wave (mmW) applications, radar, remote sensing, imaging

## Abstract

In this paper, a 3D conformal meta-lens designed for manipulating electromagnetic beams via height-to-phase control is proposed. The structure consists of a 40 × 20 array of tunable unit cells fabricated using 3D printing, enabling full 360° phase compensation. A novel automatic synthesizing method (ASM) with an integrated optimization process based on genetic algorithm (GA) is adopted here to create the meta-lens. Simulation using CST Microwave Studio and MATLAB reveals the antenna’s beam deflection capability by adjusting phase compensations for each unit cell. Various beam scanning techniques are demonstrated, including single-beam, dual-beam generation, and orbital angular momentum (OAM) beam deflection at different angles of 0°, 10°, 15°, 25°, 30°, and 45°. A 3D-printed prototype of the dual-beam feature has been fabricated and measured for validation purposes, with good agreement between both simulation and measurement results, with small discrepancies due to 3D printing’s low resolution and fabrication errors. This meta-lens shows promise for low-cost, high-gain beam deflection in mm-wave wireless communication systems, especially for sensing applications, with potential for wider 2D beam scanning and independent beam deflection enhancements.

## 1. Introduction

In numerous millimeter-wave (mmW) applications, there is always a push to enhance the performance of communication or sensing systems such as range, accuracy, frequency coverage, tunability, or other various crucial aspects that contribute to designing a fully optimized and functioning communication system [1,2,3]. In the millimeter-wave spectrum of frequencies, the design and functionality play a vital role in the system’s overall performance, because at mmW frequencies, the signals will suffer from higher propagation losses when they encounter obstacles. These losses can result in signal attenuation and reduced coverage, particularly in urban environments with dense infrastructure, meaning they are more susceptible to obstacles and even atmospheric conditions. This limits their effective range [4], making them more suitable for localized communication rather than wide-area coverage [5,6]. Incorporating the multiple input multiple output (MIMO) technology, which is a potential solution where multiple antennas are employed at both ends of the communication channel, can enhance data transmission and spectral efficiency without requiring extra bandwidth or transmission power. However, due to the close spacing of radiating elements, mutual coupling can occur in MIMO systems. Consequently, improving isolation or reducing mutual coupling between adjacent antennae presents another challenge [7]. Moreover, integrating beam deflection capabilities, especially in the millimeter-wave region, can also efficiently deliver the necessary improvements required by certain vital communication systems to our daily life [8,9].

In the realm of both present and forthcoming 5G mobile communication systems, there has been a notable surge in interest surrounding metasurfaces and metamaterials, which offer promising capabilities in molding electromagnetic radiated waves. This potential is realized through leveraging the reconfigurable characteristics of metamaterial components intelligently, such as employing refractive-index meta-lenses for the manipulation of beam shaping [10].

Metamaterials have been applied across a range of uses, such as filter-antenna sensors [11], all-metal metamaterials optimized for high-gain scenarios [12], beam-steerable frequency-selective surface (FSS)-based phase-shifting surfaces [13,14], and both ultra–wideband (UWB) and wideband antennas and filters based on FSS/EBG technology [15,16,17,18].

They typically depend on planar metamaterial components referred to as unit cells, which are considerably smaller in size and spacing compared to the wavelength, unlike antenna arrays and phased array antennas. For example, in [19], the authors proposed an electronically controlled metasurface with the ability to generate three distinct beams, i.e., Bessel, Airy, and vortex beams. Each beam is generated according to a specific scheme; for instance, when implementing the phase profile mimicking an axicon lens, a zero-order Bessel beam is generated, and so on for the other beam-manipulating schemes. Furthermore, a vortex-carrying OAM-mode smart platform, spanning from l = 0 to ±6, is also proposed in [20], where a 2-bit programmable metasurface, incorporating three PIN diodes, is proposed as an inexpensive programmable smart platform for generating the OAM beams.

Moreover, these conventional metallic meta-structures have some limitations and drawbacks in terms of size, shape, degraded performance due to conductor-induced losses, and challenges in manufacturability when unique shapes are required.

To address the complexity and cost challenges of conventional techniques, meta–structures operating in millimeter-wave frequencies can also be constructed using dielectric materials, which could offer advantages in terms of machining or fabricating complex 3D structures with the required application-wise precision. Recent progress in 3D printing dielectric materials has further expanded the possibilities, making it a viable alternative, providing both easy fabrication and high-performance meta-lenses without copper or active component-induced losses.

A key benefit offered by 3D printing lies in its capacity to produce complex structures with exceptional accuracy. This attribute proves especially advantageous in the creation of transmit arrays for millimeter-wave applications, where the precise arrangement of elements plays a crucial role in the array’s effectiveness. Through 3D printing, engineers gain the flexibility to tailor the shape, dimensions, and placement of array elements according to precise application needs. Consequently, this capability enables the optimization of both performance and efficiency in millimeter-wave transmit arrays [21]. Within the realm of this latter’s fabrication process, conventional manufacturing procedures frequently entail laborious processes, notably photo-lithography and etching, which consume considerable time and resources. Conversely, the emergence of additive manufacturing technologies, exemplified by 3D printing, introduces a transformative paradigm marked by rapid prototyping capabilities [22].

This technological advancement empowers researchers with the agility to efficiently iterate through design variations and conduct comprehensive assessments of their functional attributes and performance characteristics. Consequently, this transition engenders an expedited developmental trajectory, effectively minimizing the time required for product development and commercialization. Beyond its role in reducing time-to-market, the integration of 3D printing methodologies fosters an environment conducive to scientific exploration and innovation. Researchers are afforded the flexibility to systematically explore a diverse array of design parameters and configurations, thereby facilitating iterative experimentation and deeper insights into the complex interdependencies between structural geometries, material properties, and performance metrics. Ultimately, this comprehensive approach paves the ground for transformative breakthroughs in millimeter-wave transmit array technology, driving forward the boundaries of scientific knowledge and technological advancement [23].

Three-dimensional (3D) printing, as a versatile manufacturing method, accommodates an extensive array of materials, spanning from polymers and metals to ceramics, thus providing a spectrum of options for material selection that can be tailored to meet the specific demands of diverse applications. In the context of millimeter-wave transmit arrays, the selection of materials assumes paramount importance, particularly prioritizing those characterized by low dielectric loss and high thermal conductivity. These attributes are instrumental in mitigating signal attenuation and efficiently dissipating heat, both of which are critical considerations for ensuring optimal performance. Through meticulous selection of materials and fine-tuning of printing parameters, engineers possess the capability to customize the electromagnetic properties of the array components, thereby enhancing their suitability for millimeter-wave applications. This nuanced approach enables the optimization of performance metrics, such as signal fidelity and heat management, thus reinforcing the efficacy of millimeter-wave transmit arrays in various operational scenarios. Moreover, the synergy between material science and additive manufacturing techniques empowers engineers to continually refine and innovate in the pursuit of ever more efficient and effective solutions for millimeter-wave communication systems [24].

Recently, the focus of many researchers has been primarily on creating and enhancing both antenna and lens-based communication platforms for various applications and fields. Moreover, there are some novel promising techniques, such as artificial intelligence (AI)-based synthesizing systems, to create unique and dielectric lens designs specific to certain challenges in applications such as 5G telecommunications, satellite communications, and various sensor technologies [25,26,27,28,29]. Furthermore, reflect-array antennas are also utilized to address the aforementioned challenges associated with the utilization of the mmW spectrum, and can be achieved by using both PCB technology and 3D printing, as reported in [5], where the authors proposed a hybrid system that uses both these technologies, with the objective of creating a low cost-reflect-array system with more than 70% efficiency while operating in the mmW region. Moreover, the authors of [30] proposed a spin-selective meta-atom that is capable of absorbing incident left-handed circular polarization (LHCP) waves, converting incident right-handed circular polarization (RHCP) waves into vortex waves due to the presence of chirality resonance for the CP incident wave. On the other hand, the spin-selective meta-mirror transforms both LHCP and RHCP incident waves into OAM vortex ones at frequencies without chiral resonance, resulting in a meta-device with a variety of uses.

Several other technologies that rely on 3D-printed lenses are passive and automotive radar [31,32], satellite communication arrays for space [33,34,35,36,37], and antennas with multiple beams tailored specific 5G telecommunication systems [38,39,40,41]. It is worth mentioning that all the aforementioned applications can operate very well within millimeter-wave spectrum frequencies, owing to the practicality of dielectric lenses (no conductor losses) at these frequencies, using commercially available 3D printing technology.

Another promising area is utilizing lenses to improve the beam-steering capability for electronically steered array antennas across various applications, such as aerospace, satellite communications [42,43,44,45], automotive radar [46], and medical imaging [47,48,49].

While 3D printing holds immense promise for advancing millimeter-wave transmit arrays, several challenges remain, including material characterization, process repeatability, and scalability for mass production. Future research efforts should focus on addressing these challenges to unlock the full potential of 3D printing in millimeter-wave applications. Additionally, advancements in multi-material printing, additive manufacturing techniques, and computational design tools will further enhance the performance, efficiency, and reliability of 3D-printed transmit arrays for emerging millimeter-wave applications.

In this paper, a 3D transmitting cylindrical conformal meta-lens is proposed to realize one of many EM beam manipulating schemes, namely beam deflection, operating at 28 GHz. Height-to-phase relation is used to form the dielectric-based unit cell. The full-wave simulation environment is analyzed using CST Microwave Studio for both the unit cell design and the full beam deflection structure.

The proposed meta-lens structure is generated using a novel automatic synthesizing system with an integrated optimization process based on the genetic algorithm. This technique paved the way for tackling EM wave problems by using artificial intelligence, rendering the process of creating complex and tiresome structures relatively simple, fast, and more efficient in comparison to traditional ways. Exploring the literature, our system outperforms the traditional methods in terms of time and efficiency, in addition to the fact that our in-house-developed system is vast in functions and could incorporate other AI techniques such as deep learning and so on. The proposed automatic synthesizing method (ASM) can create a paradigm for antenna designers to tackle complex and time-consuming tasks, reducing the cost, especially when this system is utilized with affordable 3D printing technology that could eventually replace traditional technologies and predominantly solve structural complexities that exist in technologies such as PCB-based designs.

Furthermore, the simulation results clearly show the capability of the proposed 3D structure on the beam-shaping functionalities at different angles of 0°, 10°, 15°, 25°, 30°, and 45° with single- and dual-beam deflection schemes, in addition to OAM vortex generation and beam deflection. The proposed design can be further used to perform different beam-manipulating techniques in both azimuth and elevation planes.

## 2. Transmitarray Design and Analysis

### 2.1. Unit Cell Design

**Figure 1:**
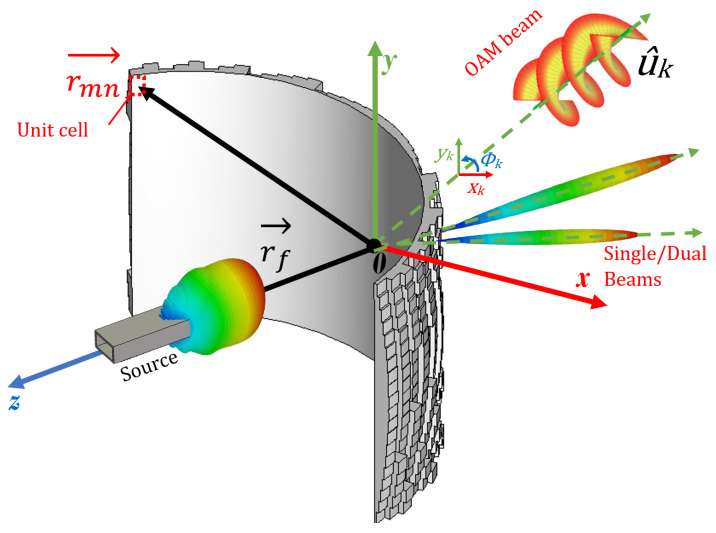
Proposed conformal 3D meta-lens with multiple beam-shaping capabilities.

Figure 1 illustrates both the OAM generation and beam deflection principles of a conformal cylindrical lens antenna. The lens is segmented into M × N sub-wavelength segments with an inner radius (R) and thickness ($h_{s}$). Using the geometric-optics method, a cylindrical wavefront can be transformed into a planar wavefront with a specified deflection angle, assuming individual control over the transmission phase of each segment (achieved in this work by adjusting the height of dielectric-based unit cells). The reference ray ${\vec{r}}_{f}$ represents the shortest ray traveling in the desired direction, and the phase difference between vector rays ${\vec{r}}_{mn}$ and ${\vec{r}}_{f}$ is expressed as [14,50]:

(1) $$\begin{array}{cc} \phi_{mn}^{c} & =-k_{0}\left|{\vec{r}}_{mn}-{\vec{r}}_{f}\right|\\ \; & +\arg\left\{\sum_{k}\exp\left[j\left(k_{0}{\vec{r}}_{mn}\cdot {\hat{u}}_{k}\pm l_{k}\Phi_{k}\right)\right]\right\} \end{array}$$

where, $k_{0}$ represents the free-space wave vector, $\phi_{mn}^{c}$ denotes the required phase of the mnth element of the meta-lens, and ${\vec{r}}_{mn}$ and ${\vec{r}}_{f}$ represent the vector positions of each unit cell and the feed antenna, respectively. Additionally, $l_{k}$ is the OAM mode number, and $\Phi_{k}$ is the azimuth angle of each unit cell in the normal plane of the proposed structure. Through compensating phase differences, the outgoing wave can be collimated to the predefined desired beam deflection direction.

The simulation considers each sub-wavelength segment as a homogeneous substrate, and the phase shift is controlled by adjusting the segment’s refractive index. The relationship between the required phase difference ($\Delta_{\varphi }$) and the refractive index difference ($\Delta_{n}$) is established as follows:

(2) $$\Delta_{\varphi }=k_{0}h_{s}\Delta_{n}$$

where $h_{s}$ is the thickness of the dielectric unit cell.

**Figure 2:**
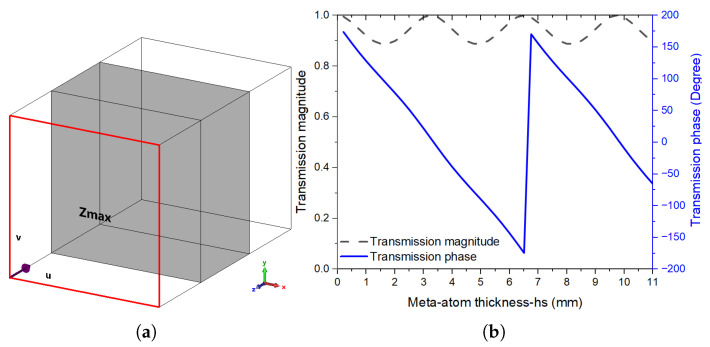
(**a**) unit cell simulation setup, and (**b**) its simulated transmission results at 28 GHz.

The simulation configuration for examining the transmission properties, both magnitude and phase, of both the refractive-based dielectric unit cell and the proposed height-to-phase-based unit cell involves utilizing periodic unit cell boundary conditions and Floquet ports, with normal incidence as illustrated in Figure 2a. The thickness of the dielectric within the unit cell serves as a parameter in the simulation setup, providing a practical avenue for designing a 3D-based meta-lens [10].

Equation (2) establishes a correlation between the thickness of the element and the phase of transmission. Leveraging previously computed phases, this relationship is established assuming a known and consistent refractive index (*n*). Consequently, it becomes feasible to fabricate the proposed meta-lens using a single dielectric slab with varying unit cell thicknesses. This methodology facilitates the incorporation of various predetermined schemes for manipulating beams, expressed as follows:

(3) $$\Delta_{h_{s}}=\Delta_{\varphi }/k_{0}\Delta_{n}$$

As $n=\sqrt{\varepsilon_{r}\mu_{r}}$, having information about the dielectric constant of the substrate is sufficient for the design and fabrication of a 3D meta-lens using available dielectric materials.

Furthermore, a dielectric unit cell based on polylactic acid (PLA) filament ($\epsilon_{r}$ = 2.72, tan δ = 0.08) is characterized in terms of height-to-phase variation, and the results are shown in Figure 2b, where it is clearly seen that the proposed unit cell exhibits high transmission amplitude, while achieving the full 360° required phase shift to almost perfectly manipulate the incident wave on the meta-lens, with various functionalities.

### 2.2. Mm-Wave 3D-Printed Lens

**Figure 3:**
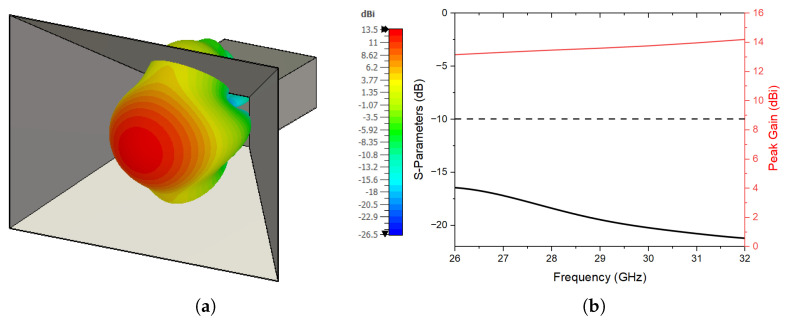
(**a**) Feeding element, and (**b**) its simulated reflection coefficient and peak gain results.

The overall proposed system consists of two main parts: the feeding source and the 3D-printed meta-lens. First, a pyramidal horn antenna with a maximum peak gain of 14.2 dBi, operating at the band of interest (26 to 32 GHz), is utilized here to illuminate the lens. Figure 3 illustrates the feeding element and its corresponding s-parameters and peak gain performances, where it is clearly seen that this kind of feeding is suitable for the proposed 3D-printed meta-lens.

In the proposed meta-lens, a PLA filament ($\epsilon_{r}$ = 2.72, tan δ = 0.08) shapes the cylindrical conformal lens with a radius (R) of 68.1 mm.

The radius value was set after calculating the minimum required number of columns to achieve the desired radiation pattern performances. In our design, our main goals were focused on having the smallest half-cylindrical lens while maintaining good performance. For instance, we took the size of a single unit cell as half the wavelength at 28 GHz, and we ran multiple full-wave simulations with different numbers of columns, with each setup having a specific radius, since this later is related to the size of the arc length, which is equal to the radius multiplied by the central angle of 180°. Calculation wise, P = 5.35 × 40 = 214 mm; L = R ·θ; R = L/θ = 68.1 mm.

The parameters of the PLA material were measured in the frequency range of 26 to 32 GHz, using the WR-28 standard waveguide-based technique incorporating the Nicolson–Ross–Weir (NRW) method described in [51]. The meta-lens is composed of a 40 × 20 array of unit cells based on the previously discussed dielectric-based unit cell, with 360° phase shift ability. Each unit cell has a size (P) of 5.35 mm, corresponding to half of the wavelength ($\lambda_{0}/2$) at 28 GHz.

All simulations were performed using both MATLAB and CST Microwave Studio, and were mainly performed at 28 GHz. They focused on the azimuth plane only for simplicity’s sake. Three distinct functionalities we designed, validated, and briefly explained in the following sections.

#### 2.2.1. Single Beam with Beam Deflection Functionality

First, three beam deflections of 0°, 15°, and 30°, respectively, were simulated using the proposed technique and were based on the previously proposed cylindrical meta-lens with a 40 × 20 array. The corresponding phase distributions of the aforementioned beam deflection angles were calculated using MATLAB and later applied as input parameters for our CST Microwave Studio-based automatic synthesizing system [52,53] to generate the proper 3D patterns.

The proposed automatic synthesizing system consists of two main sub-routines: the first part creates a Visual Basic for Applications (VBA) link between MATLAB and CST Microwave Studio. This link is critical in creating an automated system, where all tools and actions that CST has can be translated into a macro code, and this later is then transferred to MATLAB to control CST in creating simple and/or complex shapes, starting by encoding complex scripts.

Initially, the required phase profiles for the beam-manipulating schemes were calculated using Equation (1), and the preliminary results were not that optimal in terms of peak gain and side-lobe levels. To get around this, a genetic algorithm (GA)-based system is proposed for optimizing the 3D meta-lens aperture. Previous research has shown that genetic algorithms hold considerable promise for tackling electromagnetic problems, exemplified by their effectiveness in optimizing structures such as electromagnetic band gaps (EBGs), artificial magnetic conductors (AMCs), and partially reflective surfaces (PRS) [8,18,53].

**Figure 4:**
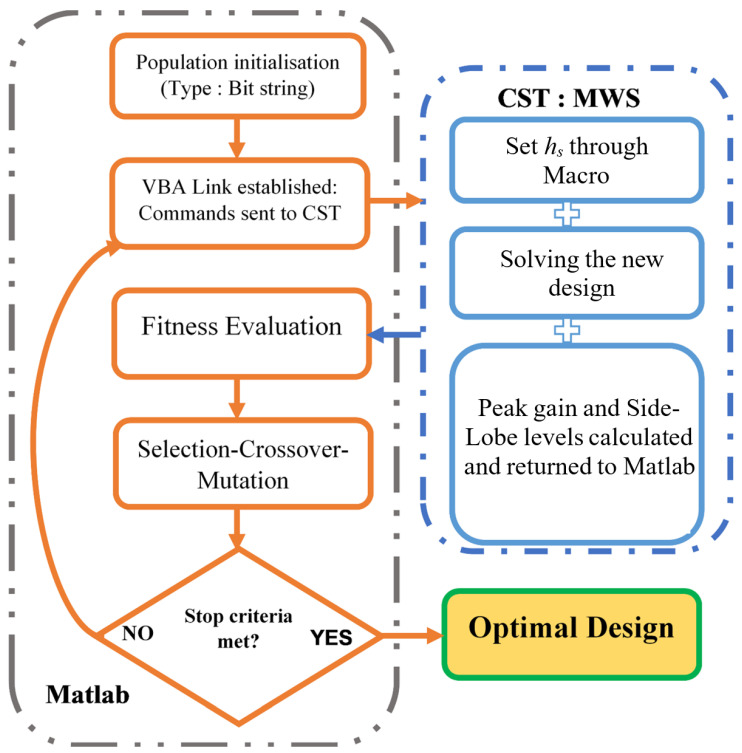
The proposed flowchart of the GA optimization process for high gain with low SLL meta-lens.

The second sub-routine of the proposed system employs MATLAB’s embedded genetic algorithm (GA) (see Figure 4). Within this optimization framework, the GA generates binary-encoded words that represent potential solutions, encompassing various compensation phases. These solutions are subjected to analysis and evaluation using a predefined fitness function, initially devised to aggregate all values exceeding 21 dBi (the initial peak gain at 28 GHz) across the frequency spectrum ranging from 26 to 32 GHz. The objective of this formulation is to design an optimized meta-lens characterized by enhanced gain with low side-lobe levels (less than −15 dB) while performing beam deflection functionality.

At first, $G_{Meta-Lens}\left(f_{i}\right)$ and $SLL(f_{i})$ are denoted as the peak gain and side-lobe level, respectively, of the 3D meta-lens structure. These values are taken at frequencies $f_{i}$, with a total of N = 13 frequency points falling within the specified overlapping frequency range of interest ($f_{min=26}$≤$f_{i}$≤$f_{max=32}$).

The optimization procedure aims to achieve two primary goals: maximizing the peak gain and minimizing SLL. These objectives are unified into a single-objective function. To facilitate this integration, weighting coefficients are allocated to the individual criteria within the main objective function [54]. The formulation is as follows:

(4) $$Fit_{Meta-Lens}=k_{1}\frac{1}{G_{Meta-Lens}\left(f_{i}\right)}+k_{2}SLL\left(f_{i}\right)$$

$k_{1}$ and $k_{2}$ are the weighting coefficients for both peak gain and SLL objectives, respectively, and are set equally to 0.5.

The optimization procedure aims to manipulate the phase of each unit cell within the frequency range of interest (26 to 32 GHz) using a genetic algorithm. The algorithm employs a uniform mutation rate of 0.001, single-point crossover, and tournament selection with a population size of 100 randomly selected binary strings representing specific phase distributions. The process involves 30 iterations, during which the top-performing structures undergo crossover and mutation until reaching the desired fitness value or reaching the maximum iteration limit.

In summary, the first sub-routine of the proposed in-house developed package deals with creating and establishing the interface between MATLAB and CST Microwave Studio, and the second one handles the optimization process. The first sub-routine process takes only 30 s to create the whole lens (40 × 20 array). As for the case of the second one, it takes from 2 h to 12 h in total after reaching the desired results or when the number of generations has been met during the process of the genetic algorithm. Each full-wave simulation takes around 15 min using a computer station with Intel® Core™ i7-13700HX with a clocking speed of 2.10 GHz, 64 GB of RAM, and GPU computing enabled on an RTX 4070 with 8 GB of GDDR6.

The final phase distributions optimized by the genetic algorithm at 28 GHz for 0°, 15°, and 30° are attained after the 15th iteration when the termination criterion is met.

**Figure 5:**
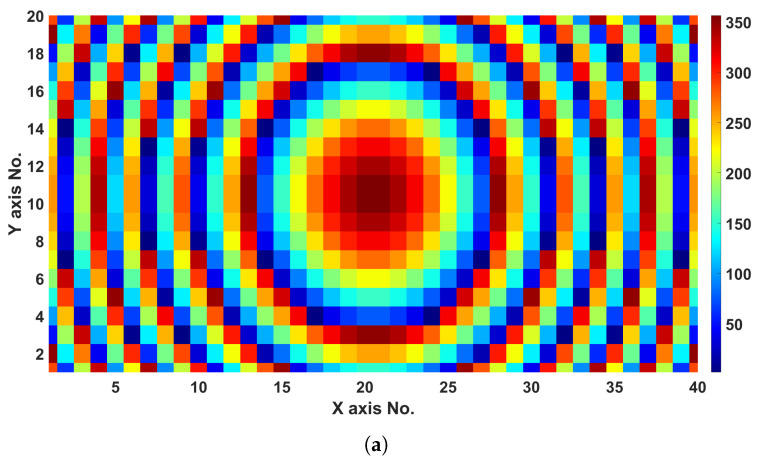

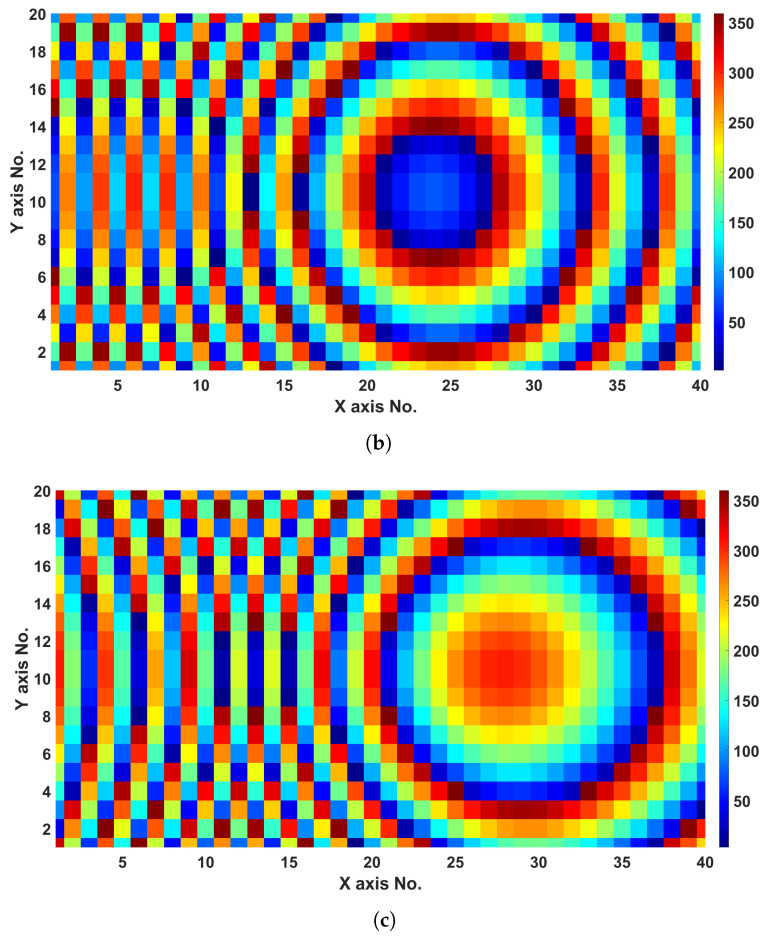
Beam deflection phase distribution at 28 GHz: (**a**) 0°, (**b**) 15°, and (**c**) 30° schemes.

**Figure 6:**
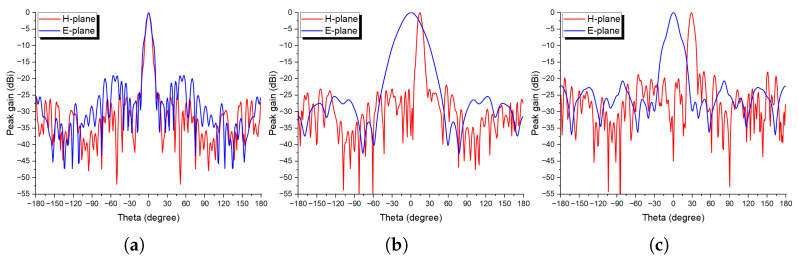
Beam deflection performance at 28 GHz (**a**) 0°, (**b**) 15°, and (**c**) 30° in both H- and E-planes.

Figure 5 depicts the generated phase distribution for the targeted angles. Full-wave simulations were carried out with the final optimized designs for three different beam deflection angles, and the results are illustrated in Figure 6. Maximum peak gains of 25.9, 25.5, and 23.8 dBi for the 0°, 15°, and 30° at 28 GHz, so almost invariant with the beam scanning, with only 2.1 dBi loss at 30°. Moreover, the side lobe level at 0° was −24.6 dB; however, it increased with the beam deflection by 2.7 and 6.7 dB at 15° and 30°, respectively.

#### 2.2.2. OAM Vortex Generation

As is widely known, the current communication systems have certain limitations in terms of bandwidth (BW) and signal-to-noise ratio (SNR), which hinder the ability of existing antennas to meet the escalating data rate requirements for today’s users. As a result, there is a critical necessity for the development of alternative approaches that can fulfill users’ expectations for connectivity. Orbital angular Momentum (OAM) antennas have emerged as promising candidates to address these challenges.

In contrast to conventional antennas, OAM antennas operate by transmitting signals through their phase fronts. Each mode of an OAM antenna possesses a distinct helical phase front that is orthogonal to other modes, facilitating the independent transmission of multiple data streams. Unlike the conventional method where multiple signals necessitate dividing the available bandwidth into separate sections, OAM antennas offer a unique advantage. They have the capability to generate multiple modes from a single aperture, leveraging this characteristic to enhance channel capacity and consequently boost data rates. This distinctive feature underscores the potential of OAM antennas in meeting the evolving demands of wireless communication systems.

To that extent, another unique beam manipulation scheme has been realized, which involves converting the main beam into an OAM beam of arbitrary mode (in this case l = +1) and steering this later on to any predefined angles.

**Figure 7:**
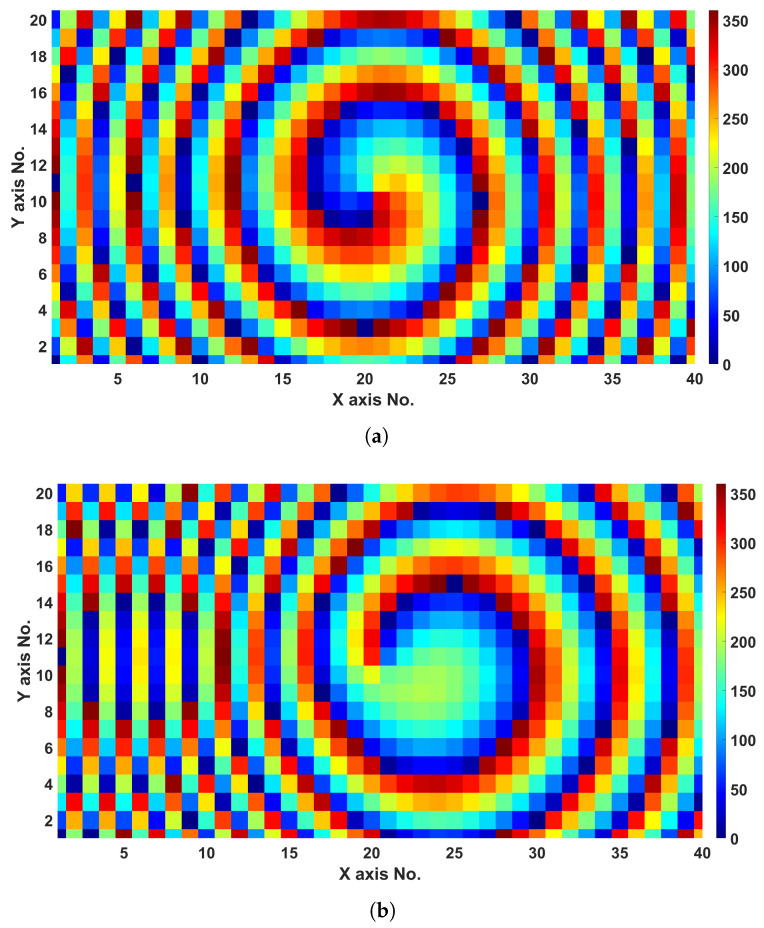

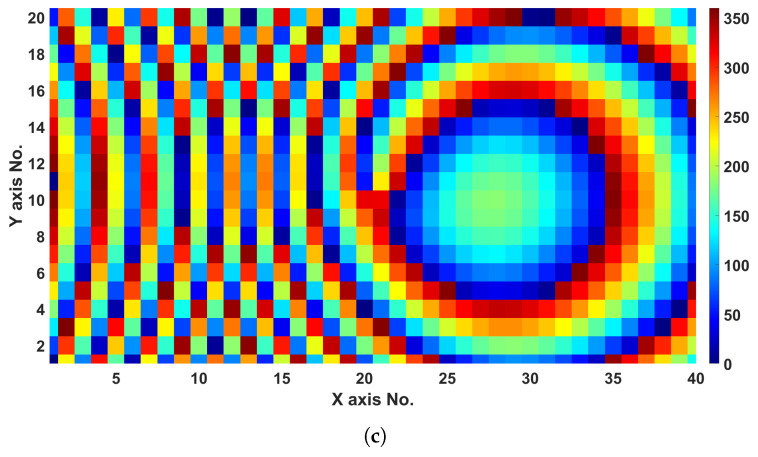
OAM Mode l = +1 phase distributions at 28 GHz, for (**a**) 0°, (**b**) 15°, and (**c**) 30° beam deflecting schemes.

**Figure 8:**
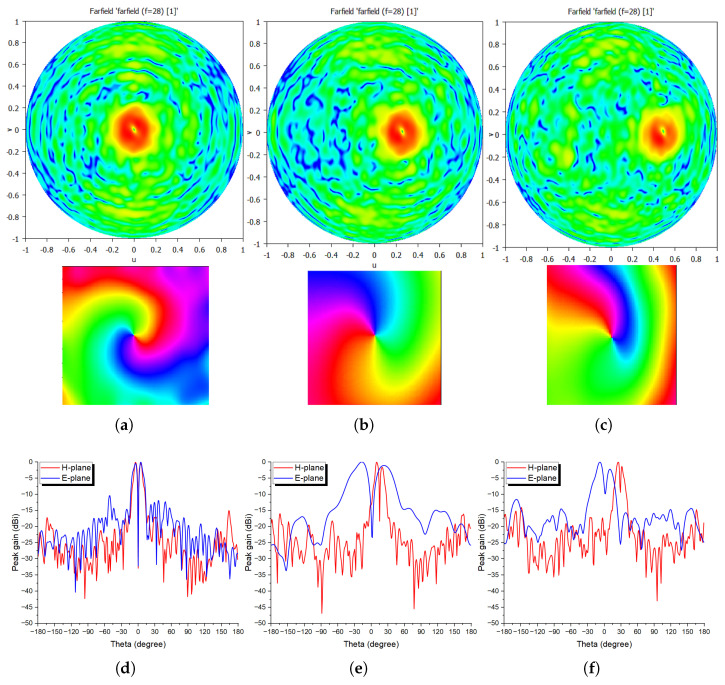
OAM Mode l = +1 amplitude and phase responses at 28 GHz, for (**a**) 0°, (**b**) 15°, and (**c**) 30°; (**d**–**f**) their corresponding normalized radiation patterns in both H- and E-planes.

Similar to the previous feature, the required phase distributions for both generating the OAM mode and also deflecting it, are calculated using the same MATLAB-to-CST Microwave synthesizing system, and are depicted in Figure 7. Both amplitude and phase response of these three OAM beams are presented, with both OAM null and spiral phase are seen in Figure 8a–c, proving the feasibility of the proposed 3D-printed meta-lens in both generating and deflecting OAM waves.

The maximum achieved peak gain at 28 GHz, for 0°, 15° and 30°, are 20.2, 19.9, and 19.8 dBi, so almost invariant with the beam scanning, where only 0.4 dBi loss when 30° deflection is chosen.

#### 2.2.3. Dual Beam with Beam Deflection Functionality

**Figure 9:**
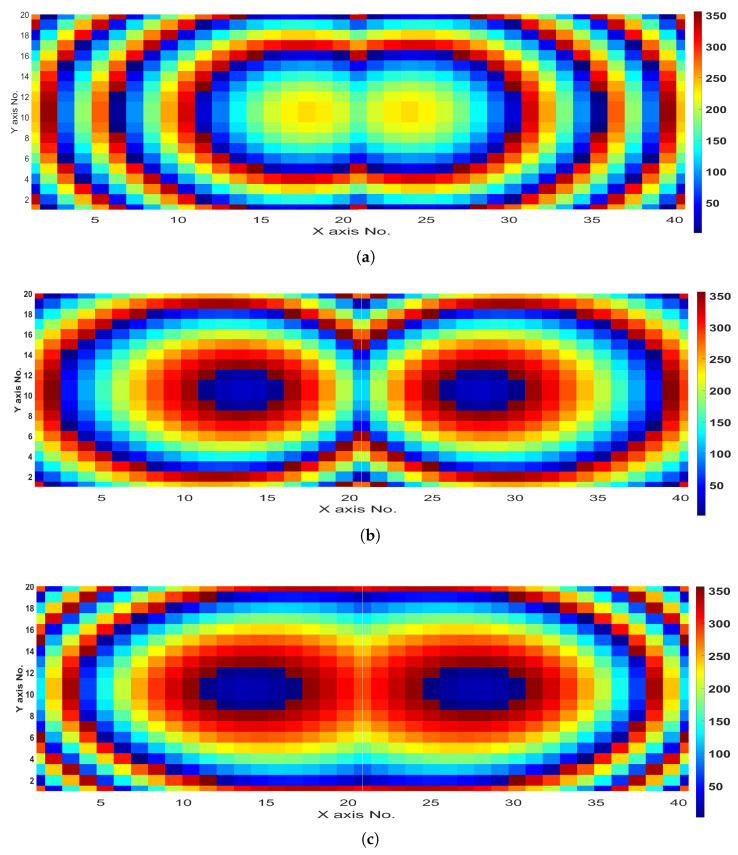
Beam-splitting (dual-beams) phase distributions at 28 GHz: (**a**) 10°, (**b**) 25°, and (**c**) 45° schemes.

**Figure 10:**
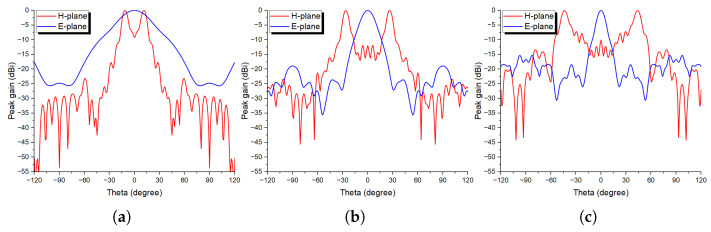
Beam-splitting (dual-beams) normalized radiation patterns at 28 GHz, for (**a**) 10°, (**b**) 25°, and (**c**) 45° schemes, for both H- and E-plane cuts.

Another interesting beam manipulating scheme is also designed, where the main beam is split into two beams steered simultaneously at different predefined angles. Figure 9 depicts the calculated required phase distributions for three different beam deflection angles of 10°, 25°, and 45°, respectively. The resulting normalized radiation patterns are illustrated in Figure 10, where both H- and E-planes are considered at 28 GHz. It can be noted that the beams are almost perfectly in accordance with the desired beam deflection angles. This kind of feature can be useful for point-to-multipoint communication applications, without the need for two transmitters or more, and can simultaneously broadcast in different directions with dual or even more beams, depending on the required links between the transmitting device and the receiving counterparts. Moreover, independent beam deflection for different beams can be attained using the proposed method, and can also be extended to work in both azimuth and elevation planes as per the application’s requirements.

## 3. Fabrication and Measurement Results

A 40 × 20 dielectric-based conformal prototype of the previously proposed dual-beam lens, designed for −45° and 45° angles, was optimized and fabricated on PLA filament ($\epsilon_{r}$ = 2.72, tan δ = 0.08) using 3D printing technology.

The process of fabrication involves defining multiple parameters as inputs in our in-house developed software. After fixing those parameters, by setting the desired 3D printing material, and by calculating the required thicknesses of each unit cell and optimizing it through the genetic algorithm, a phase distribution corresponding to a specific beam deflection function is generated and automatically sent from MATLAB R2021a to CST to auto-synthesize and generate the final optimized full 3D lens. This latter is then bent using the bending function of the CST Microwave Studio Academic version while respecting the number of unit cells and the focal distance (or the radius in this case). All these steps are performed automatically thanks to the well-developed in-house software. Finally, the full-wave simulation is carried out to confirm the correctness of the desired beam deflection angle or function, and then the final STEP file of the design is exported to the dedicated 3D printing software to prepare it for fabrication.

The effective aperture of the final fabricated lens comprises 800 elements. A linear–polarized horn antenna serves as a source to illuminate the aperture, with a focal distance of F = 68.1 mm.

**Figure 11:**
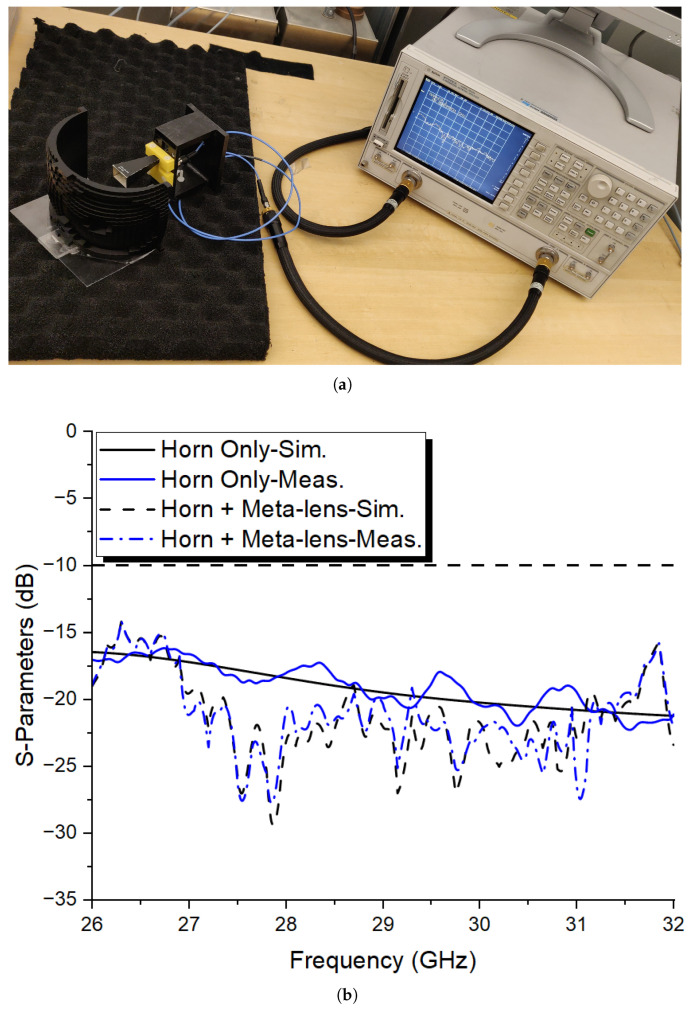
(**a**) Proposed 3D-printed meta-lens for dual-beam shaping with its VNA measurement setup; (**b**) simulated versus measured reflection coefficient results with and without the meta-lens.

First, the reflection coefficient evaluation of the proposed antenna, with and without the 3D conformal meta-lens structure, is carried out using an Agilent 8722ES vector network analyzer (VNA), as shown in Figure 11a. The measurement results show that the proposed 3D meta-lens antenna covers the band of interest, spanning from 26 to 32 GHz, with less than -15 dB in reflection coefficient.

It is also compared to the simulated results, where the proposed antenna operates at the same band of frequencies. Therefore, both simulation and measurement results are in good agreement despite the slight discrepancies between them, which are probably due to assembly and fabrication errors. Moreover, by using a dielectric-based lens, the reflection coefficient is not affected much, as seen in Figure 11b, which is another advantage compared to a copper-/dielectric-based transmit array.

**Figure 12:**
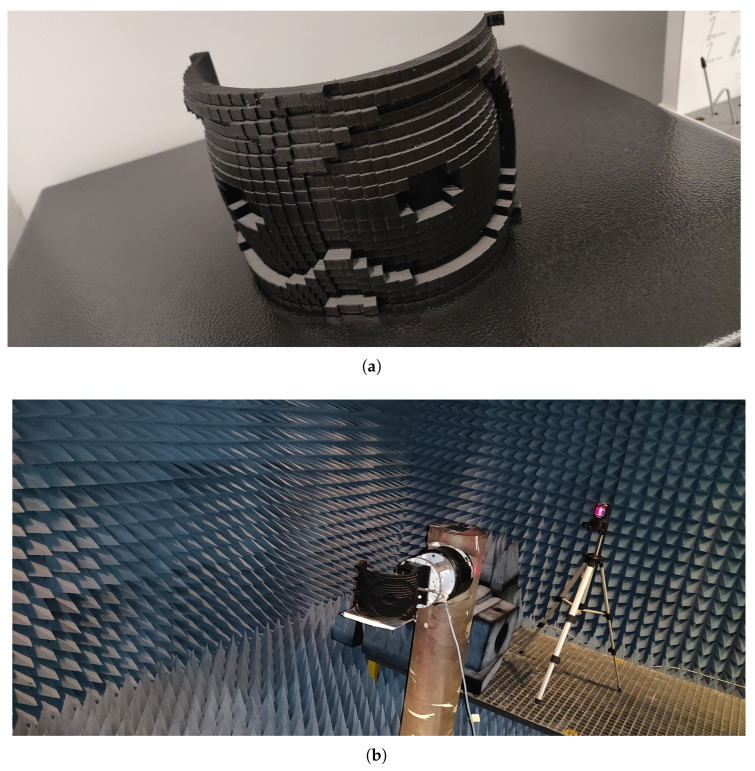
(**a**) Proposed 3D-printed meta-lens for dual-beam shaping and (**b**) its measurement setup in the anechoic chamber.

For further analysis, the fabricated meta-lens underwent measurements for peak gain and radiation patterns in the far-field region. The measurements were conducted in an anechoic chamber setup, as depicted in Figure 12b.

**Figure 13:**
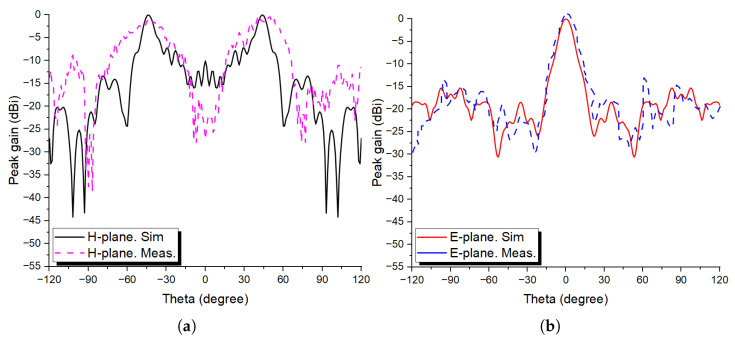
Simulated versus measured normalized radiation patterns for the dual-beam prototype in both (**a**) H- and (**b**) E-plane cuts at 28 GHz.

The simulated versus measured normalized radiation patterns in both plane cuts are depicted in Figure 13, where it is clearly seen that both simulation and measurement results agree well with some discrepancies due to fabrication errors such as the discontinuities between the unit cells in both directions (column and row wise) and the inadequate and insufficient resolution during the 3D printing process, which affected the overall performance of the fabricated prototype. Moreover, the simulated peak gain was 18.5 dBi, whereas in the case of measurement, the maximum attained peak gain was 15.1 dBi, so a loss of almost 3.4 dBi is noted.

Despite these discrepancies, the proposed automatic synthesizing system proved to be effective in designing 3D-printed meta-lenses with versatile features and capabilities, with lower cost and ease of fabrication compared to other technologies. Furthermore, there are no copper or conductor losses associated with 3D printing technology, and if combined with the emerging RF 3D printable materials, such as Radix Printable Dielectric [55], more efficient and better-performing meta-lenses can be designed at higher mm-wave frequencies.

For the sake of simplicity, all beam deflection schemes are only implemented at the azimuth plane, and this can be extended to a 2D beam-deflecting environment with independent dual-beam deflection functionalities, proving the versatility of the presented meta-lens for future mm-wave applications.

## 4. Conclusions

This study introduced a transmissive meta-lens featuring a core component known as the 3D dielectric unit cell. This unit cell achieved complete 360° phase compensation at 28 GHz while maintaining a minimum transmission amplitude of 0.9. This capability established a height-based tunable unit cell with minimal loss, ideal for shaping electromagnetic waves for various applications. Moreover, a genetic algorithm was used with the proposed automatic synthesizing system for both creating and optimizing the final structure. Furthermore, the proposed design enabled the creation of three distinct beam deflection systems at 28 GHz. These systems include highly focused beams with varying deflection angles, generation of orbital angular momentum (OAM), and dual beams with beam deflection capabilities. The results demonstrated exceptional performance in terms of beam scanning and functional versatility. With its cost-effectiveness and adaptability, the 3D-printed structure holds promise as a candidate for high-gain and beam-shaping applications in future millimeter-wave wireless sensing systems. 

## Data Availability

The original contributions presented in the study are included in the article, further inquiries can be directed to the corresponding author.
